# Primary cutaneous CD4+ small/medium T-cell lymphoproliferative disorder: a case with multiple tumors^⋆^

**DOI:** 10.1016/j.abd.2022.05.008

**Published:** 2023-05-22

**Authors:** Esra Sarac, Cuyan Demirkesen

**Affiliations:** a Department of Dermatology, Koc University School of Medicine, Istanbul, Turkey; b Department of Pathology, Acibadem Mehmet Ali Aydinlar University School of Medicine, Istanbul, Turkey

Dear Editor,

Primary cutaneous CD4+ small/medium T-cell lymphoproliferative disorder (PCSM-TCLPD) is defined to be 6% of primary cutaneous lymphomas.^1^ Although it is considered to have an uncertain malignant potential, it has almost always a benign prognosis with locally limited indolent tumors. Due to the benign biological behavior, World Health Organization’s working group renamed and classified this entity as a lymphoproliferative disorder in 2016, and it was suggested to no longer diagnose this provisional entity as an overt lymphoma.^2^

## Case report

**Figure 1:**
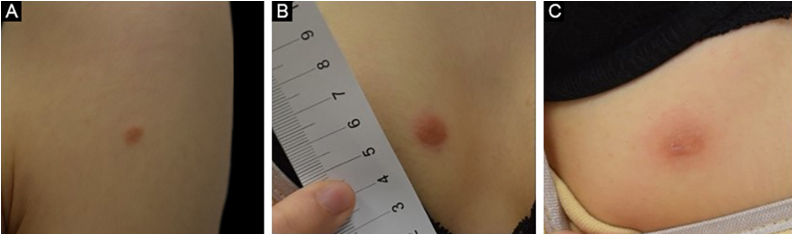
Clinical appearance of the lesions. Erythematous nodules on (A) left deltoid, (B) right pectoral, and (C) right abdominal region

A 25-year-old female patient was referred to the dermatology department with the complaint of slowly enlarging masses on her chest, abdomen, and upper arm for the past year. The lesions were slightly itchy. She had been applying mild topical corticosteroids to the lesions without any improvement. She had no medical history or concomitant disease. On physical examination, there were erythematous firm nodules sized approximately 0.5‒1 cm in diameter located on the left deltoid (Fig. 1A), right pectoral (Fig. 1B), and right abdominal area (Fig. 1C).

**Figure 2:**
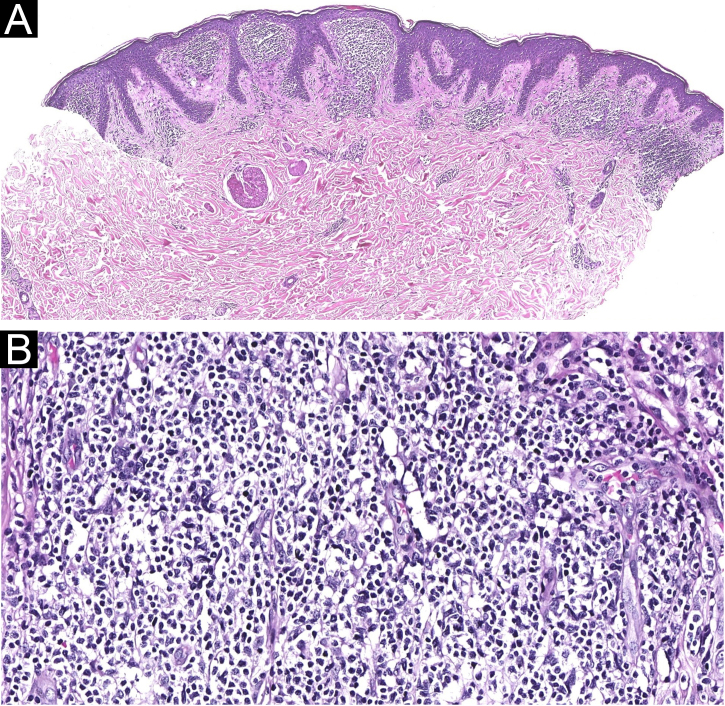
(A) Diffuse, dense infiltrate filling the papillary dermis, without prominent epidermotropism (Hematoxylin & eosin, ×20). (B) Small to medium-sized lymphoid cells, with mild pleomorphism (Hematoxylin & eosin, ×200)

**Figure 3:**
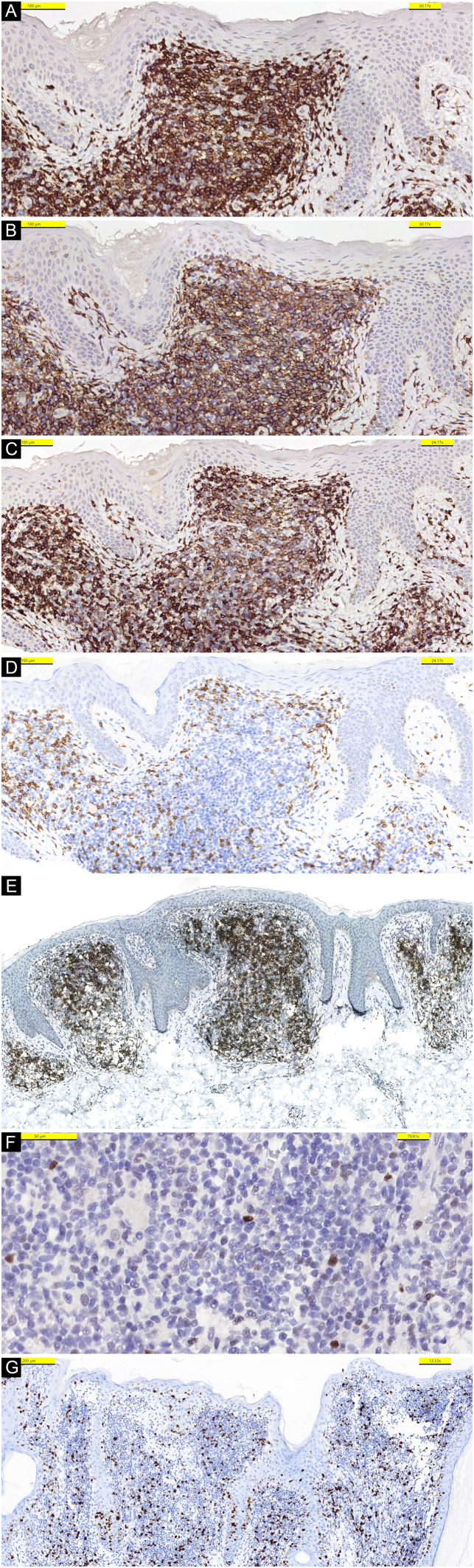
Lymphoid cells expressing CD3 (A), CD4 (B), CD7 (C), but negative for CD8 (D). (E) High intensity immunostaining with PD-1. (F) Weak staining with Bcl-6. (G) Ki-67 activation

A punch biopsy was performed on the abdominal nodule. Histopathological examination revealed dense, diffuse infiltration within the papillary dermis, composed of small and medium-sized T-lymphoid cells, with pleomorphic nuclei (Fig. 2). There are only a few large cells in the dermal infiltrate. Epidermotropism was seen in only the focal area. The lymphocytes were CD3+ (Fig. 3A), CD4+ (Fig. 3B), CD5+, CD7+ (Fig. 3C), CD8- (Fig. 3D), CD30-.

Stainings with PD-1 (Fig. 3E) and bcl-6 (Fig. 3F) were detected. CD8+ cells represent a reactive minor population. Reactive infiltrate of CD20+ B-cells was also present. Proliferative activity with Ki-67 was detected as approximately 10%‒15% in the infiltration (Fig. 3G). Based on these histopathological and immunological findings, the tumor was diagnosed as PCSM-TCLPD.

Due to the localization and multiplicity of the lesions, and also considering the aesthetic concern, a non-aggressive treatment option was preferred in this case. Oral doxycycline 100 mg per day was administered to our patient. Regression of the lesions started in the third week of the treatment and a total clinical remission was achieved after 4 months. She has been in our follow-up for 6 months after cessation of doxycycline and no recurrence has been observed.

## Discussion

PCSM-TCLPD is a provisional entity with a benign prognosis. The classical clinical presentation of PCSM-TCLPD is an asymptomatic solitary erythematous papule, nodule, or plaque. Itching, pain, and ulceration have occasionally been defined. Multiple lesions as in our case have been described in the literature.^3^ The clinical course is variable, and lesions may wax and wane. The most common localizations are the head, neck, upper extremities, and trunk.^4^, ^5^ It is frequently observed in adults, however, pediatric cases have also been reported.^6^ Histopathologically, tumors mostly consist of a dense infiltration of small to medium-sized pleomorphic T-cells with mild to moderate atypia. Tumor cells are often accompanied by B-cells, plasma cells, histocytes, and eosinophils. The tumoral infiltrate may have a band-like or nodular appearance in the dermis. Epidermotropism can be seen in focal areas. Tumor cells are CD3 positive, CD4 positive, CD30 negative; and also, follicular helper T-cell markers Programmed Death-1 (PD-1), B-Cell Lymphoma-6 (BCL-6), C-X-C motif Chemokine Ligand-13 (CXCL-13) and Inducible T-cell Costimulator (ICOS) are positive, immunophenotypically.^3^, ^7^, ^8^ There are no clinical or histological definitive criteria for diagnosing an aggressive pattern of the tumor.

Pseudolymphoma, primary cutaneous follicle center lymphoma, primary cutaneous marginal zone lymphoma, and tumoral stage of mycosis fungoides (MF) should be considered clinically in the differential diagnosis. Due to the clinical and histopathological overlap, the most difficult one to distinguish from PCSM-TCLPD could be pseudolymphoma. Causes and triggers of pseudolymphoma such as insect bites, infections, drugs and foreign agents (tattoo, piercing, etc.) should be questioned. For MF, the existence of patches and plaques belonging to different stages of the disease, and epidermotropism of lymphocytes with atypical cerebriform nuclei in the histopathological examination are used to distinguish the disease from PCSM-TCLPD.

There is no standard guideline for the treatment, options can be assessed according to individual features of the cases. Several reports showed that spontaneous resolution is possible after biopsy, therefore conservative approach rather than aggressive treatments is preferable. The modalities found to be successful in the treatment are topical or intralesional steroids, excision, radiotherapy, and systemic doxycycline.^9^, ^10^ The prognosis is excellent with a 100% 5-year disease-specific survival rate.^1^

## Financial support

None declared.

## Authors’ contributions

Esra Sarac: Study concept and design, data collection, analysis and interpretation of data, writing of the manuscript, critical review of the literature, final approval of the final version of the manuscript.

Cuyan Demirkesen: Study concept and design, data collection, analysis and interpretation of data, writing of the manuscript, final approval of the final version of the manuscript.

## Conflicts of interest

None declared.
